# Supplementary material for: Hepatitis C virus NS3 helicase contributes to (−) strand RNA synthesis
Source: Nat Commun. 2025 Aug 27;16:8006. doi: 10.1038/s41467-025-63498-9 (PMC12391449; doi:10.1038/s41467-025-63498-9)
Supplement: Supplementary file 1 — Supplementary Information [file 41467_2025_63498_MOESM1_ESM.pdf]

Supplementary Material

Hepatitis C virus NS3 helicase contributes to (-) strand RNA synthesis

Philipp Ralfs[1], Stéphane Bressanelli[2], Lina M. Günter[3], Alexander Gabel[3,4], Paul Rothhaar[1], Kyle J. Price[5], Thibault Tubiana[2], Mathias Munschauer[1,3,4], David N. Frick[5], Volker Lohmann*[1]

[1]Department Infectious Diseases, Molecular Virology, Heidelberg University, Medical Faculty, Heidelberg, Germany; [2]Université Paris-Saclay, CEA, CNRS, Institute for Integrative Biology of the Cell (I2BC), 91198, Gif-sur-Yvette, France; [3]Helmholtz Institute for RNA-based Infection Research (HIRI), Helmholtz-Center for Infection Research (HZI), Würzburg, Germany; [4]Institute of Medical Virology, University Hospital Frankfurt, Goethe University, Frankfurt am Main, Germany; [5]University of Wisconsin, Milwaukee, USA;

* To whom correspondence should be addressed. Tel: +49 (0)6221-56 6449; Fax: +49 (0)6221-56 4570; Email: Volker.lohmann@med.uni-heidelberg.de

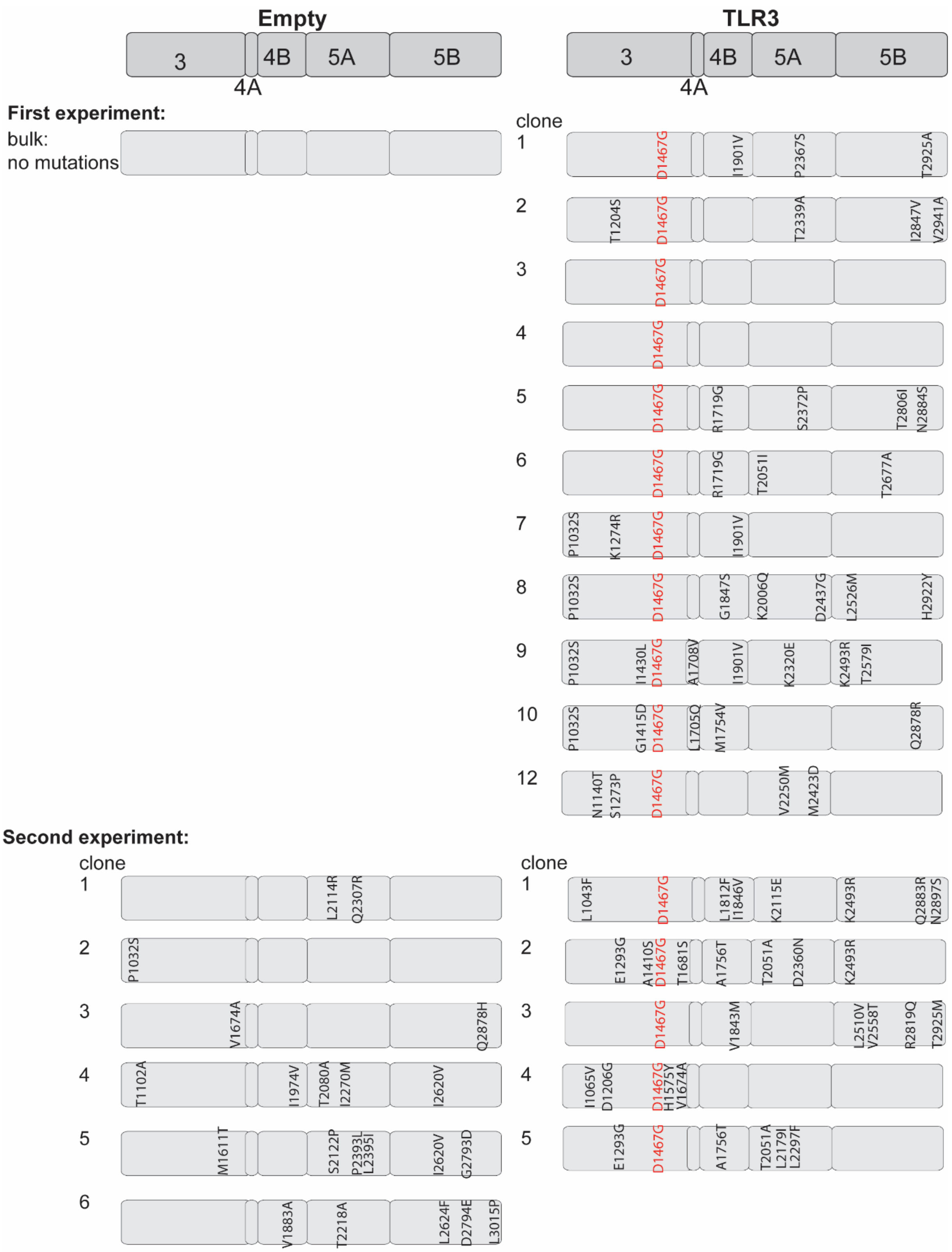

Supplementary Figure 1: D1467G is selected under TLR3 expression. Sequencing results of directed evolution approach of two independently conducted experiments. The HCV NS3-5B coding sequence was amplified from total RNA of a bulk population (empty cells, exp. 1) or from 5-12 cell clones. PCR products were subjected to direct Sanger-sequencing and optimized automatic base-calling was performed by Microsynth Seqlab. Coding mutations are indicated at their approximate location in the viral genome. The only mutation present in all TLR3-transduced clones is highlighted in red (D1467G).

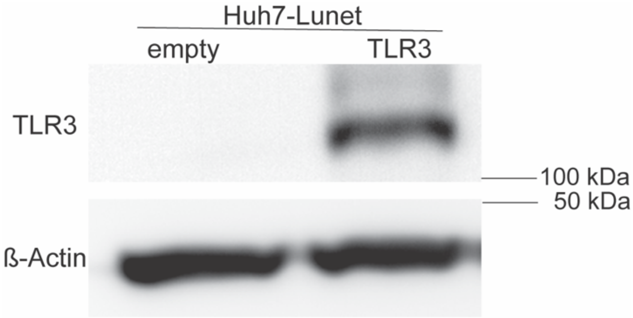

Supplementary Figure 2: TLR3 expression is detectable in Huh7 Lunet TLR3 but not in empty control cells. Whole cell lysates of Huh7-Lunet empty and TLR3 were analysed regarding TLR3 and ß-actin expression by WB. Representative of n=3 independent biological replicates is shown.

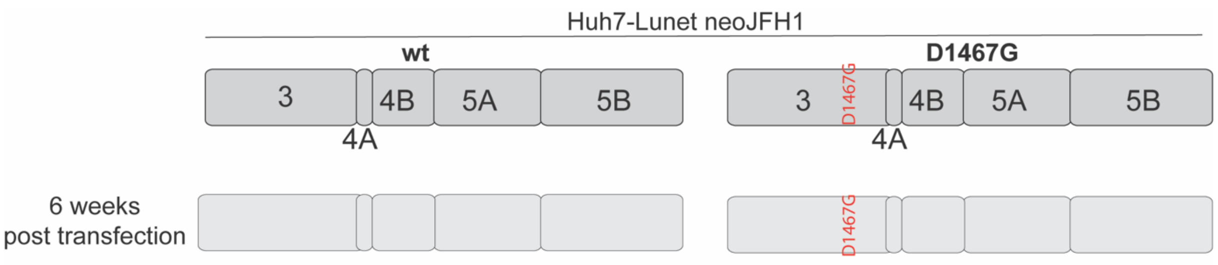

Supplementary Figure 3: Long-term passaging of Huh7 Lunet neoJFH1 D1467G does not lead to reversion of D1467G or appearance of other dominant mutations. Cells were harvested at least six weeks post electroporation and subjected to RT-PCR and Sanger sequencing. Optimized automatic base-calling was performed by Microsynth Seqlab. D1467G is indicated at its respective position in the viral genome and highlighted in red. Representative of n=3 independent biological replicates is shown.

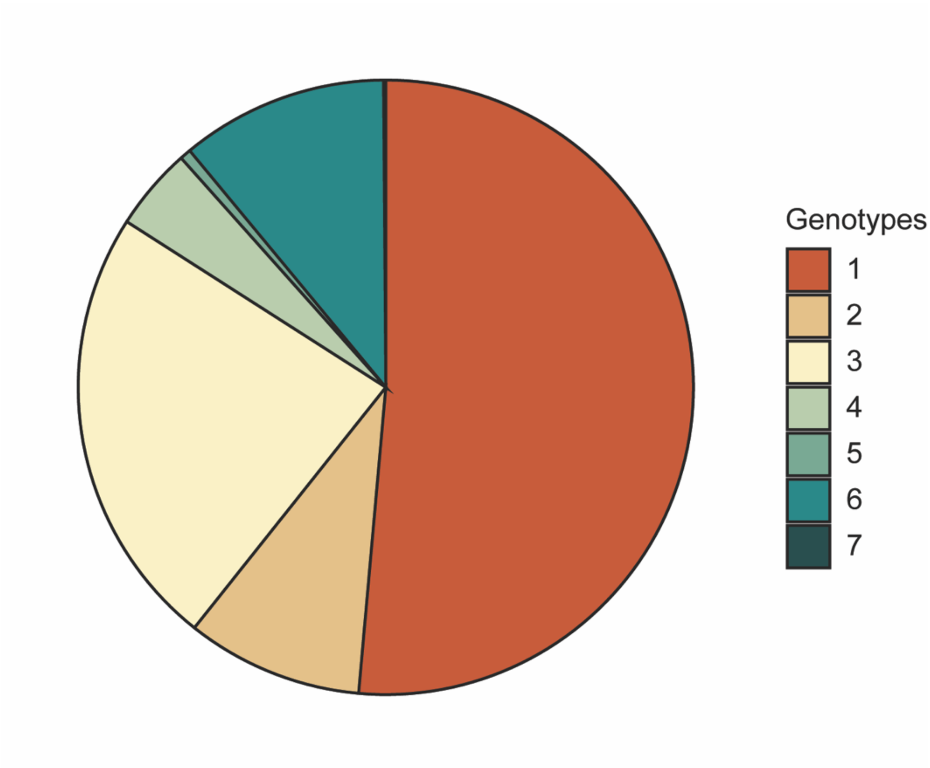

Supplementary Figure 4: Genotypes of 2555 full-length HCV genomes in the GLUE database. Genotype information was retrieved from GLUE database.

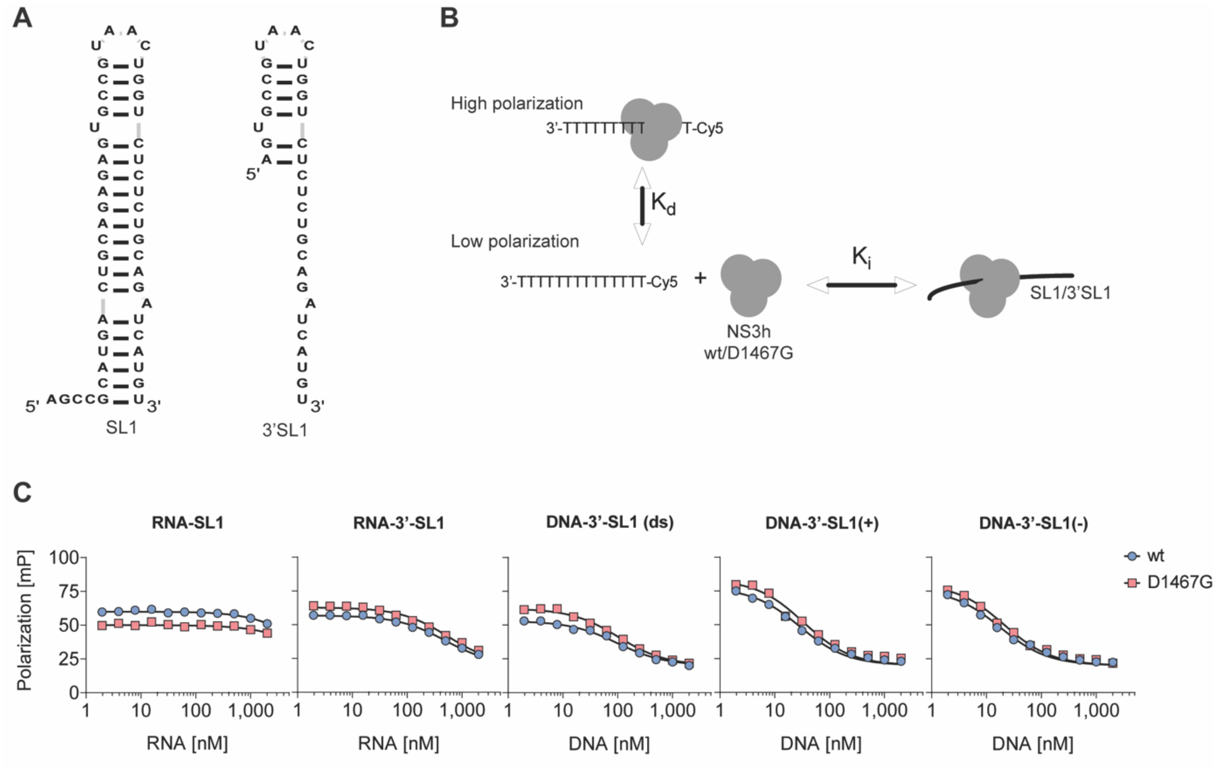

D

DNA/RNA	K_i [nM] wt	K_i [nM] D1467G
RNA-SL1	>1500	>1500
RNA-3'-SL1	121±13	137±28
DNA-3'-SL1(ds)	25±5	25±4
DNA-3'-SL1(+)	7.1±2	8.3±5
DNA-3'-SL1(-)	4.4±1	5.2±2

Supplementary Figure 5: D1467G does not affect binding of NS3 to 3'X SL1. A) Illustration of 3'X SL1 sequences used in Fluorescence polarization (FP)-based DNA- and RNA-binding assays. B) Scheme illustrating FP-based binding assays as described previously (Mukherjee et al., 2012). Fluorescence helicase substrate Cy5-TTTTTTTTTTTTTTT-3' (Cy5-dT15) binds to NS3h, altering its polarization. Binding of SL1 oligonucleotides to NS3h wt/D1467G alters interaction of NS3h with Cy5-dT15 and therefore affect measured polarization, with effect dependent on interaction strength and concentration of oligonucleotide present. C) Measured polarization in the presence of indicated SL1 RNA or DNA oligonucleotides. Mean of three independent experiments are shown. D) Ki [nM] values calculated from three independent experiments. Data were fit assuming the probe binds with a K_d of 5 nM and that the other NAs act as competitors.

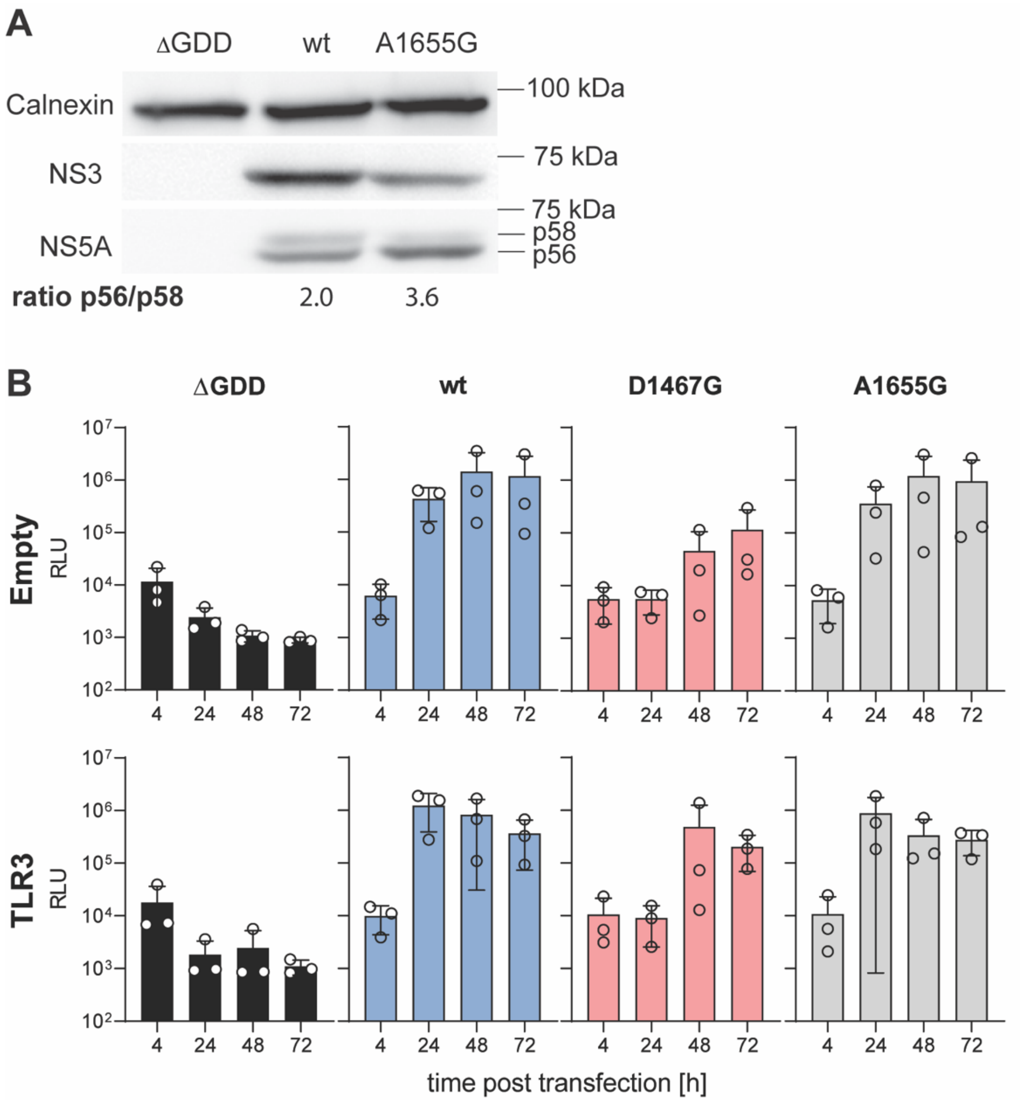

Supplementary Figure 6: NS5A hyperphosphorylation is not required for TLR3 activation. A-B) Huh7-Lunet cells were electroporated with ivt RNA of JFH1 wt/D1467G/A1655G subgenomic replicon. A) Effect on NS5A hyperphosphorylation was analysed by WB and p56/p58 ratio was quantified. Representative of three independent biological replicates. B) Replication at indicated time points was monitored using luciferase reporter activity (n=3 independent biological replicates). Mean values are indicated and standard deviation is indicated by error bars.

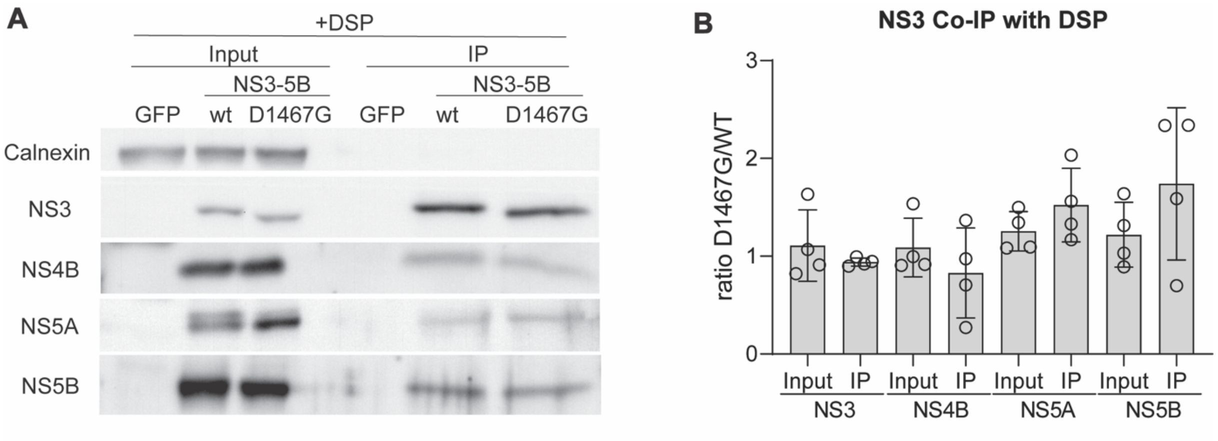

Supplementary Figure 7: NS3 interaction with NS4B, NS5A and NS5B is detectable upon intracellular crosslinking. A-B) JFH1 NS3-5B wt/D1467G was transiently expressed in Huh7-Lunet T7 cells and cells were treated with the crosslinker Dithiobis-Succinimidylpropionat (DSP) 30 min prior to lysis as described in the Supplementary Materials and Methods section. A) NS3 was immunoprecipitated and Co-IP of other HCV NS proteins was analysed by WB. Band intensity was measured using FIJI. Representative of four independent replicates shown. B) Ratio of respective D1467G to wt band (n=4 independent biological replicates). Statistical analysis was performed using two-sided unpaired t-test using Graph Pad Prism. P-values are indicated. Mean values are indicated and standard deviation is indicated by error bars.

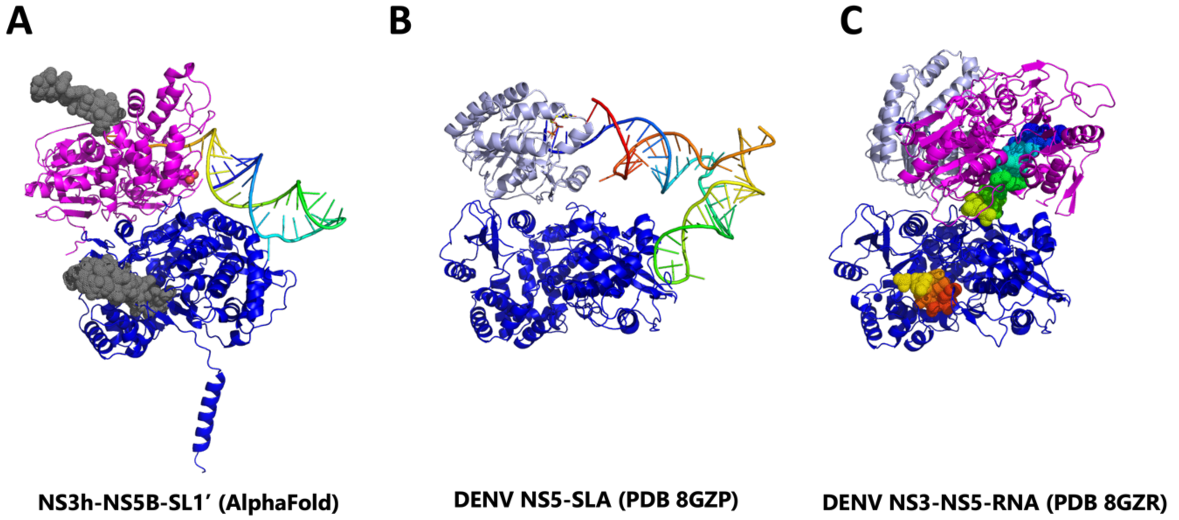

Supplementary Figure 8: Side-by-side comparison of the putative HCV NS3h-NS5B-SL1′ priming complex and of the cryo-electron microscopy structures of dengue virus replicase complexes. A) HCV NS3h-NS5B-SL1′ model as in Fig. 9, except that the extra single-stranded RNA bases added to highlight the exit of RNA from NS3h and the NS5B template binding groove are now displayed as gray spheres. B) NS5 complex with DENV stem-loop A. The polymerase domain is colored blue and in the same orientation as HCV NS5B. The N-terminal methyltransferase domain is light blue and SLA in rainbow colors from blue (5′ end) to red (3′ end). C) DENV NS3-NS5 complex with RNA. NS3 is colored magenta and the single-stranded RNA coming out of it and into the NS5 polymerase template binding groove is displayed as spheres colored from blue (5′) to red (3′).

Supplementary Materials and Methods

Reagents

Supplementary Table 1: Oligonucleotides used in this study

**Name**	**Sequence 5′-3′**
**Cloning**	
D1467G_1_fwd	GCCCAGAAGGTACCCCATTG
D1467G_1_rev	GGCTGAAGCCGACAGCTTGG
D1467G_2_fwd	CCAAGCTGTCGGCTTCAGCC
D1467G_2_rev	CAGCTTGCATGCATGTGGC
GLT1E_D1463G_fwd1	GTAACGAGCTCGCCGCGCAGCTG
GLT1E_D1463G_rev1	GCTGAAACCGACTGTCTGGATGAC
GLT1E_D1463G_fwd2	GTCATCCAGACAGTCGGTTTCAGC
GLT1E_D1463G_rev2	CTCGACGAGGTCGACGTCCG
A1655G_1_rev	CCTCAAGGTCACCTTGCATGCAT
A1655G_2_fwd	ATGCATGCAAGGTGACCTTGAGG
A1655G_2_rev	CTTGATGTGGTTAACGGCCCTC
G2U_fwd	CAG ATC ATT TGG CCG GCA TGG TCC CAG C
G2U_rev	CAT GCC GGC CAA ATG ATC TGC AGA GAG ACC AGT TAC GGC
**RT and Replicase amplification**	
A9466-MluI (rev)	AGCTATGGAGTGTACCTAGTGTGTGCC
S EMCV-2	CGTCTAGGCCCCCCGAAC
A9482 (RT)	GGAACAGTTAGCTATGGAGTGTACC
**Sequencing**	
S/2A/4053	GGTACTTGCATGCTCCAACTGGCAG
S/2A/4808	CACAAGACGCTGTCTCACGCAGTC
S/2A/5677	CTTCATTAGCGGCATCCAATACCTC
S/2A/6478	CATCTCTGGCAATGTCCGCCTGGG
S/2A/7350	GAGAGCACCATATCAGAAGCC
S/2A/7673	ATACCACCGTGTGCTGCTCCATGT
S/2A/8083	ACACCAATTCCCACAACCATCATG
S/2A/8503	CAAACCTGCGGTTACAGACGTTGC
S/2A/8913	GGCTGGGAAACATCATCCAGTATGC
A/2A/7860	GCTTGATGTCCTTTAAGACTGAGTC
**qPCR**	
IFIT1_fwd	GAAGCAGGCAATCACAGAAA
IFIT1_rev	TGAAACCGACCATAGTGGAA
GAPDH_fwd	GAAGGTGAAGGTCGGAGTC
GAPDH_rev	GAAGATGGTGATGGGATTTC
JFH1_fwd	TCTGCGGAACCGGTGAGT
JFH1_rev	GGGCATAGAGTGGGTTTATCCA
JFH1_probe	AAAGGACCCAGTCTTCCCGGCAATT
**ATPase assay**	
dCT	CTCTCTCTCTCTCTCTCT
dGA	GAGAGAGAGAGAGAGAGA

dU18	UUUUUUUUUUUUUUUUUU
pppRNA	pppAGGGCUGCGGGCCUGCGGCCUGCCAUGGCC
Poly(U) RNA	Sigma (Catalog number P9528)
**Binding assays**	
RNA-SL1	pppAGCCGCAUGACUGCAGAGAGUGCCGUAACUGGUCUCUCUGCAGAUCAUGU
RNA-3'SL1	pppAGUGCCGUAACUGGTCUCUCUGCAGAUCAUGU
DNA-3'SL1(+)	CAGTAATACGACTCACTATTAGTGCCGTAACTGGTCTCTCTGCAGATCATGT
DNA-3'SL1(-)	ACATGATCTGCAGAGAGACCAGTTACGGCACTAATAGTGAGTCGTATTACTG
DNA-SL1(+)	CAGTAATACGACTCACTATTAGCCGCATGACTGCAGAGAGTGCCGTAACTGGTCTCTCTGCAGATCATGT
DNA-SL1(-)	ACATGATCTGCAGAGAGACCAGTTACGGCACTCTCTGCAGTCATGCGGCTAATAGTGAGTCGTATTACTG

Supplementary Table 2: Antibodies used in this study

Target	Species, clonality, isotype	WB dilution	IF dilution	Vendor/ Reference	Article number
NS3	Mouse monoclonal "2E3", IgG1	1:3000	1:1000	BioFront technologies	HCV-2E3
NS3	Rabbit polyclonal "49", IgA purified	1:1000	n.a.	[1]	n.a.
NS4B	Polyclonal rabbit serum	1:500	n.a.	[1]	n.a.
NS5A	Mouse monoclonal "9E10", IgG2	1:3000	n.a.	[2]	n.a.
NS5A	Rabbit polyclonal serum "52"	1:500	n.a.	[1]	n.a.
NS5B	Rabbit polyclonal serum	1:200	n.a.	[1]	n.a.
dsRNA	Mouse monoclonal "J2", IgG2a	n.a.	1:250	Jena bioscience	RNT-SCI-10010500
Calnexin	Rabbit polyclonal	1:2000	n.a.	Enzo	ADI-SPA-860
MAVS	Rabbit polyclonal "AT107"	1:1000	n.a.	Enzo	ALX-210-929-C100
ß-Actin	Mouse monoclonal IgG1	1:4000	n.a.	Sigma-Aldrich	A5441
TLR3	Rabbit monoclonal "D10F10"	1:2000	n.a.	Cell Signaling	6961

Supplementary Table 3: Enzymes, kits, Chemicals

Name	Vendor	Catalog number
Calf intestinal phosphatase (CIP)	New England Biolabs, Ipswich, USA	M0525L
Anti-Digoxigenin-AP, Fab-Fragment	Roche, Mannheim, Germany	11093274910
DIG washing and blocking buffer for Northern blot	Roche, Mannheim, Germany	11585762001
CSPD, ready-to-use AP substrate	Roche, Mannheim, Germany	CSPD-RO
DNaseI	Promega, Madison, USA	M6101
Easy Hyb hybridization solution	Roche, Mannheim, Germany	11603558001
Dynabeads protein G	Thermo Fisher Scientific, Waltham, USA	10003D
Gibson assembly master mix (2x)	New England Biolabs, Ipswich, USA	E2611L
NucleoBond PC500	Macherey-Nagel, Düren, Germany	740574.50
NucleoSpin Plasmid	Macherey-Nagel, Düren, Germany	740588.50
NucleoSpin RNA	Macherey-Nagel, Düren, Germany	740984.50
NucleoSpin Gel and PCR purification	Macherey-Nagel, Düren, Germany	740609.250
Nylon membrane, positively charged, for Northern blot	Roche, Mannheim, Germany	11 417 240 001
RNAsin	Promega, Madison, USA	N2511
T3 RNA polymerase	Promega, Madison, USA	P2083
T4 DNA ligase	Thermo Fisher Scientific, Waltham, USA	M0202S
T7 RNA polymerase	Produced in house (ref)	n.a.
Transit-LT1 transfection reagent	Mirus Bio, Madison, USA	MIR2304
Phusion Flash High-Fidelity PCR Master mix	Thermo Fisher Scientific, Waltham, USA	F548L
High-Capacity cDNA Reverse Transcription Kit	Thermo Fisher Scientific, Waltham, USA	4368813
qScript XLT One-Step RT-qPCR Mix	Qanta Biosciences, Gaithersburg, USA	95132-100
2x iTaq Universal SYBR Green Mix	Bio-Rad, Munich, Germany	1725125
dsRNA bioassay	Promega, Madison, USA	W2041

Supplementary Table 4: Specialized commercial instruments

Name	Vendor
CFX96 Touch Real-Time PCR Detection System	Bio-Rad, Munich, Germany
Gene Pulser II electroporation system	Bio-Rad, Munich, Germany
Berthold Luminometer Microplate reader LB 940	Berthold Technologies, Bad Wildbad, Germany
Cell discoverer 7 (CD7) widefield microscope	Carl Zeiss Microscopy, Jena, Germany

Web Sites/Data Base Referencing

Supplementary Table 5: Software and programs

Name	Vendor/Reference	Version
AlphaFold3	https://alphafoldserver.com/	n.a.
Bio-Rad CFX Maestro	Bio-Rad, Munich, Germany	2.0
Pymol	Schrödinger Inc., New York, USA	3.1
FIJI	Fiji.sc[3]	2.9
Cellpose	Cellpose.org[4]	1
GraphPad Prism	GraphPad Software	9
Adobe Illustrator	Adobe	27.1.1
Benchling	Benchling	n.a.

Fluorescence polarization (FP)-based DNA- and RNA-binding assay

SL1 RNAs were transcribed from annealed (+) and (-) sense oligonucleotides (Supplementary Table 1) using the HiScribe T7 High Yield RNA Synthesis Kit (New England Biolabs). T7 Gene 2.5 promoter sequences are shown in grey. RNA was purified using the PureLink RNA Mini Kit (Invitrogen).

Binding of SL1-derived RNA and DNA oligonucleotides to NS3h wt/D1467G was analysed by measuring the polarization of fluorescently labelled oligonucleotide probe as described previously [5]. Assays were performed in low-volume 384-well black microplates (Greiner Bio-One, #784076). Each oligonucleotide was diluted in an 11-point 2-fold series starting at 20 µM. From each dilution, 2 µL was transferred to 18 µL aliquots of a solution containing NS3h, the Cy5-dT15 probe (Cy5-TTTTTTTTTTTTTTT-3'), and binding buffer. After transfer, final concentrations were 15 nM NS3h wt/D1467G, 15 nM Cy5-dT15, 25 mM MOPS, pH 7.5, 1.25 mM

$MgCl_2$, 0.0025 mg/ml BSA, 0.005% (v/v) Tween20 and 0.025 mM DTT. SL1-based RNA or DNA oligonucleotides were present at concentrations ranging from 0-2,000 nM to each well. After 20 min at 23 °C, polarization was analysed using a TECAN Infinite M1000 Pro multi-mode microplate reader by exciting at 635nm and measuring total fluorescence intensity, parallel and perpendicular polarized light at 667nm. The data were fit to Eq. S1, where B_{max} is the polarization in the absence of competing oligonucleotide, [Cy5] is the concentration of Cy5-dT15 probe (15 nM), K_d of dissociation constant of the probe and each enzyme (5 nM), [NA] is the concentration of competing nucleic acids. G-factors were calculated using a solution of 15 nM Cy5-dT15 in the absence of NS3h, which was normalized to a value of 20 mP.

$$P = \frac{B_{Max}[Cy5]}{K_d\left(1+\frac{[NA]}{K_i}\right)+[Cy5]} + 20 \quad \text{(S2)}$$

Crosslinking Co-Immunoprecipitation

For crosslinking, cells were treated prior to lysis with Pierce Dithiobis-Succinimidylpropionat (DSP), a thiol-cleavable, primary amine-reactive, cell-permeable crosslinker. Therefore, 1mg Pierce DSP (Thermo Fisher Scientific) was freshly reconstituted with 100µL of DMSO to create a 25mM solution. Cells were washed twice with PBS and incubated with 1.25mM DSP in PBS for 30min at room temperature. The crosslinking reaction was quenched by incubation with 20mM Tris pH7.5, for 15min at room temperature. Afterwards, samples were processed as described for non-crosslinked samples.

References

1. Backes, P. *et al.* Role of annexin A2 in the production of infectious hepatitis C virus particles. *J Virol* **84**, 5775-5789 (2010). https://doi.org/10.1128/JVI.02343-09
2. Lindenbach, B. D. *et al.* Complete replication of hepatitis C virus in cell culture. *Science* **309**, 623-626 (2005). https://doi.org/10.1126/science.1114016
3. Schindelin, J. *et al.* Fiji: an open-source platform for biological-image analysis. *Nat Methods* **9**, 676-682 (2012). https://doi.org/10.1038/nmeth.2019
4. Stringer, C., Wang, T., Michaelos, M. & Pachitariu, M. Cellpose: a generalist algorithm for cellular segmentation. *Nat Methods* **18**, 100-106 (2021). https://doi.org/10.1038/s41592-020-01018-x
5. Mukherjee, S. *et al.* Identification and analysis of hepatitis C virus NS3 helicase inhibitors using nucleic acid binding assays. *Nucleic Acids Res* **40**, 8607-8621 (2012). https://doi.org/10.1093/nar/gks623
