# Supplementary material for: Hepatitis C virus NS3 helicase contributes to (−) strand RNA synthesis
Source: Nat Commun. 2025 Aug 27;16:8006. doi: 10.1038/s41467-025-63498-9 (PMC12391449; doi:10.1038/s41467-025-63498-9)
Supplement: Supplementary file 3 — Description of Additional supplementary files [file 41467_2025_63498_MOESM3_ESM.pdf]

File Name: Supplementary Data 1
Description: Significantly enriched NS3 wt and D1467G binding sites in (+) and (-) HCV RNA with (adjusted $p<0.05$). Statistical test: One-sided Fisher's exact test, Benjamini-Yekutieli procedure was applied to correct for multiple testing.

File Name: Supplementary Data 2
Description: Significantly enriched NS3 wt and D1467G binding sites in host RNA with (adjusted $p<0.05$). Statistical test: One-sided Fisher's exact test, Benjamini-Yekutieli procedure was applied to correct for multiple testing.

File Name: Supplementary Data 3
Description: Cif files containing the AF3 prediction of the 5BSL3-NS3h and the SL1-NS5B-NS3h complex shown in Figure 9 and Supplementary Figure 8.
