# Supplementary material for: Hepatitis C virus NS3 helicase contributes to (−) strand RNA synthesis
Source: Nat Commun. 2025 Aug 27;16:8006. doi: 10.1038/s41467-025-63498-9 (PMC12391449; doi:10.1038/s41467-025-63498-9)
Supplement: Supplementary file 7 — Reporting Summary [file 41467_2025_63498_MOESM7_ESM.pdf]

nature portfolio

Corresponding author(s): Prof. Dr. Volker Lohmann

Last updated by author(s): Aug 5, 2025

Reporting Summary

Nature Portfolio wishes to improve the reproducibility of the work that we publish. This form provides structure for consistency and transparency in reporting. For further information on Nature Portfolio policies, see our Editorial Policies and the Editorial Policy Checklist.

Statistics

For all statistical analyses, confirm that the following items are present in the figure legend, table legend, main text, or Methods section.

n/a	Confirmed	
☐	☒	The exact sample size (*n*) for each experimental group/condition, given as a discrete number and unit of measurement
☐	☒	A statement on whether measurements were taken from distinct samples or whether the same sample was measured repeatedly
☐	☒	The statistical test(s) used AND whether they are one- or two-sided<br>*Only common tests should be described solely by name; describe more complex techniques in the Methods section.*
☒	☐	A description of all covariates tested
☒	☐	A description of any assumptions or corrections, such as tests of normality and adjustment for multiple comparisons
☐	☒	A full description of the statistical parameters including central tendency (e.g. means) or other basic estimates (e.g. regression coefficient) AND variation (e.g. standard deviation) or associated estimates of uncertainty (e.g. confidence intervals)
☐	☒	For null hypothesis testing, the test statistic (e.g. *F*, *t*, *r*) with confidence intervals, effect sizes, degrees of freedom and *P* value noted<br>*Give P values as exact values whenever suitable.*
☒	☐	For Bayesian analysis, information on the choice of priors and Markov chain Monte Carlo settings
☒	☐	For hierarchical and complex designs, identification of the appropriate level for tests and full reporting of outcomes
☒	☐	Estimates of effect sizes (e.g. Cohen's *d*, Pearson's *r*), indicating how they were calculated

Our web collection on statistics for biologists contains articles on many of the points above.

Software and code

Policy information about availability of computer code

Data collection	AlphaFold3 for structural modelling;
Data analysis	Cellpose for cellular segmentation, FIJI for analysis of fluorescent intensity and preparation of representative images; GraphPad Prism (V9) for graphing and statistical analysis; Benchling for vector analysis, cloning strategy development and sequence alignment; Bio-Rad CFX Maestro for analysis of RT-qPCR data;<br>The computer code for the custom analyses is publicly available at https://github.com/AlexGa/cRIP-eCLIP-workflow as of the date of publication.

For manuscripts utilizing custom algorithms or software that are central to the research but not yet described in published literature, software must be made available to editors and reviewers. We strongly encourage code deposition in a community repository (e.g. GitHub). See the Nature Portfolio guidelines for submitting code & software for further information.

Data

Policy information about availability of data

All manuscripts must include a data availability statement. This statement should provide the following information, where applicable:

- Accession codes, unique identifiers, or web links for publicly available datasets
- A description of any restrictions on data availability
- For clinical datasets or third party data, please ensure that the statement adheres to our policy

Next-generation sequencing data have been deposited to GEO and can be accessed with the accession number GSE292413 and reviewer token qnobmgsmxnmpdsr. The remaining data underlying this article are available in the article and in its online supplementary material. Source Data are provided with this paper.

Research involving human participants, their data, or biological material

Policy information about studies with human participants or human data. See also policy information about sex, gender (identity/presentation), and sexual orientation and race, ethnicity and racism.

Reporting on sex and gender	not applicable
Reporting on race, ethnicity, or other socially relevant groupings	not applicable
Population characteristics	not applicable
Recruitment	not applicable
Ethics oversight	not applicable

Note that full information on the approval of the study protocol must also be provided in the manuscript.

Field-specific reporting

Please select the one below that is the best fit for your research. If you are not sure, read the appropriate sections before making your selection.

☒ Life sciences ☐ Behavioural & social sciences ☐ Ecological, evolutionary & environmental sciences

For a reference copy of the document with all sections, see nature.com/documents/nr-reporting-summary-flat.pdf

Life sciences study design

All studies must disclose on these points even when the disclosure is negative.

Sample size	We performed at least n=3 independent biological replicates for all experiments. This adheres to common standards of good scientific practice.
Data exclusions	No data were excluded from the analyses
Replication	All attempts of replication were successful.
Randomization	This is not relevant to our study since all experiments are built on distinct constructs and mutants.
Blinding	Experiments are built on distinct constructs and mutants. Therefore, blinding is not relevant or possible in this setting.

Reporting for specific materials, systems and methods

We require information from authors about some types of materials, experimental systems and methods used in many studies. Here, indicate whether each material, system or method listed is relevant to your study. If you are not sure if a list item applies to your research, read the appropriate section before selecting a response.

Materials & experimental systems

n/a	Involved in the study
	☒ Antibodies
	☒ Eukaryotic cell lines
☒	Palaeontology and archaeology
☒	Animals and other organisms
☒	Clinical data
☒	Dual use research of concern
☒	Plants

Methods

n/a	Involved in the study
☒	ChIP-seq
☒	Flow cytometry
☒	MRI-based neuroimaging

Antibodies

Antibodies used

HCV NS3 Mouse monoclonal "2E3", IgG1 BioFront technologies HCV-2E3
HCV NS3 Rabbit polyclonal "49", IgA purified; Backes et al., 2010;
HCV NS4B Polyclonal rabbit serum; Backes et al., 2010;
HCV NS5A Mouse monoclonal "9E10", IgG2; Lindenbach et al., 2005;
HCV NS5A Rabbit polyclonal serum "52"; Backes et al., 2010;
HCV NS5B Rabbit polyclonal serum; Backes et al., 2010;
dsRNA Mouse monoclonal "J2", IgG2a Jena bioscience RNT-SCI-10010500
Calnexin Rabbit polyclonal Enzo ADI-SPA-860
MAVS Rabbit polyclonal "AT107" Enzo ALX-210-929-C100
ß-Actin Mouse monoclonal IgG1 1:4000 Sigma-Aldrich A5441
TLR3 Rabbit monoclonal "D10F10" 1:2000 Cell Signaling 6961

Validation

Western blotting and immunofluorescence with cells not expressing HCV antigens (Backes et al., 2005; Lindenbach et al., 2005), Biofront technologies, Figure 3) or TLR3 (Supplementary Figure 2)

Eukaryotic cell lines

Policy information about cell lines and Sex and Gender in Research

Cell line source(s)

Grunvogel, O. et al. Secretion of Hepatitis C Virus Replication Intermediates Reduces Activation of Toll-Like Receptor 3 in Hepatocytes. Gastroenterology 154, 2237-2251 e2216 (2018). https://doi.org/10.1053/j.gastro.2018.03.020
Schaller, T. et al. Analysis of hepatitis C virus superinfection exclusion by using novel fluorochrome gene-tagged viral genomes. J Virol 81, 4591-4603 (2007). https://doi.org/10.1128/JVI.02144-06
Friebe, P., Boudet, J., Simorre, J. P. & Bartenschlager, R. Kissing-loop interaction in the 3' end of the hepatitis C virus genome essential for RNA replication. J Virol 79, 380-392 (2005). https://doi.org/10.1128/JVI.79.1.380-392.2005
Bender, S. et al. Activation of Type I and III Interferon Response by Mitochondrial and Peroxisomal MAVS and Inhibition by Hepatitis C Virus. PLoS Pathog 11, e1005264 (2015). https://doi.org/10.1371/journal.ppat.1005264
Koutsoudakis, G., Herrmann, E., Kallis, S., Bartenschlager, R. & Pietschmann, T. The level of CD81 cell surface expression is a key determinant for productive entry of hepatitis C virus into host cells. J Virol 81, 588-598 (2007). https://doi.org/10.1128/JVI.01534-06
Backes, P. et al. Role of annexin A2 in the production of infectious hepatitis C virus particles. J Virol 84, 5775-5789 (2010). https://doi.org/10.1128/JVI.02343-09

Authentication

Multiplex human cell line authentication test

Mycoplasma contamination

negative

Commonly misidentified lines
(See ICLAC register)

no commonly misidentified cell lines have been used in this study

Plants

Seed stocks	*Report on the source of all seed stocks or other plant material used. If applicable, state the seed stock centre and catalogue number. If plant specimens were collected from the field, describe the collection location, date and sampling procedures.*
Novel plant genotypes	*Describe the methods by which all novel plant genotypes were produced. This includes those generated by transgenic approaches, gene editing, chemical/radiation-based mutagenesis and hybridization. For transgenic lines, describe the transformation method, the number of independent lines analyzed and the generation upon which experiments were performed. For gene-edited lines, describe the editor used, the endogenous sequence targeted for editing, the targeting guide RNA sequence (if applicable) and how the editor was applied.*
Authentication	*Describe any authentication procedures for each seed stock used or novel genotype generated. Describe any experiments used to assess the effect of a mutation and, where applicable, how potential secondary effects (e.g. second site T-DNA insertions, mosiacism, off-target gene editing) were examined.*
